# DNA Assembly‐Based Stimuli‐Responsive Systems

**DOI:** 10.1002/advs.202100328

**Published:** 2021-05-14

**Authors:** Shasha Lu, Jianlei Shen, Chunhai Fan, Qian Li, Xiurong Yang

**Affiliations:** ^1^ School of Chemistry and Chemical Engineering Frontiers Science Center for Transformative Molecules Institute of Translational Medicine Shanghai Jiao Tong University Shanghai 200240 China; ^2^ Institute of Molecular Medicine Shanghai Key Laboratory for Nucleic Acid Chemistry and Nanomedicine Department of Urology Renji Hospital School of Medicine Shanghai Jiao Tong University Shanghai 200127 China

**Keywords:** biomedical applications, dynamic DNA nanotechnology, nanofabrication, stimuli‐responsive systems

## Abstract

Stimuli‐responsive designs with exogenous stimuli enable remote and reversible control of DNA nanostructures, which break many limitations of static nanostructures and inspired development of dynamic DNA nanotechnology. Moreover, the introduction of various types of organic molecules, polymers, chemical bonds, and chemical reactions with stimuli‐responsive properties development has greatly expand the application scope of dynamic DNA nanotechnology. Here, DNA assembly‐based stimuli‐responsive systems are reviewed, with the focus on response units and mechanisms that depend on different exogenous stimuli (DNA strand, pH, light, temperature, electricity, metal ions, etc.), and their applications in fields of nanofabrication (DNA architectures, hybrid architectures, nanomachines, and constitutional dynamic networks) and biomedical research (biosensing, bioimaging, therapeutics, and theranostics) are discussed. Finally, the opportunities and challenges for DNA assembly‐based stimuli‐responsive systems are overviewed and discussed.

## Graphical abstract

Various DNA assembly‐based stimuli‐responsive systems can be designed and fabricated based on different exogenous stimuli, including DNA strand, pH, light, temperature, electricity, metal ions, etc. The response mechanisms of DNA assembly‐based stimuli‐responsive systems as well as their application in nanofabrication and biomedical research are summarized. The current challenges and future potential are also discussed.

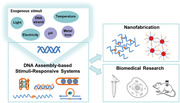

## 1 Introduction

The establishment of the Watson–Crick double helix model promoted the understanding of DNA structures to a new stage, and the robust Watson–Crick base pairing between adenine (A), thymine (T), guanine (G), and cytosine (C) led to the development of autonomous synthetic DNA.^[^ ^1^ ^]^ As a consequence, the DNA nanotechnology has revolutionized bottom‐up nanofabrication by taking DNA out of their biological environment and using their information for precise assembly.^[^ ^2^, ^3^, ^4^ ^]^ With the rapid development of DNA nanotechnology, it has been found that DNA is neither static nor just exists in conventional duplex form. DNA sequences can fold into different nanostructures under certain conditions: the self‐complementary sequences can form the hairpin structure,^[^ ^5^ ^]^ guanine‐rich sequences can fold into G‐quadruplex structure in the presence of metal ions (e.g., Na^+^, K^+^, Pb^2+^),^[^ ^6^, ^7^ ^]^ cytosine‐rich sequences can assemble into i‐motif structure under acidic pH,^[^ ^8^, ^9^ ^]^ the formation T–A•T and C–G•C^+^ through Hoosteen base pairing forms the basis of the triplex structure,^[^ ^10^, ^11^, ^12^ ^]^ the discovery of ion‐bridged complexes (T–Hg^2+^–T/C–Ag^+^–C) provides the possibility for the duplex structure of mismatched DNA sequences.^[^ ^13^, ^14^ ^]^ Beyond the structure diversity, a variety of nucleic acids have sequence specificity, for example, RNA‐cleaving DNAzymes can respond to specific metal ions,^[^ ^15^, ^16^ ^]^ and the aptamer can bind its target specifically.^[^ ^17^, ^18^ ^]^ The thermodynamic and electronegativity properties of DNA, the response of G‐quadruplex to metal ions, the response of i‐motif and triplex structure to pH values, the response of metal ion bridged complexes to Hg^2+^ or Ag^+^, and the response of RNA‐cleaving DNAzymes to specific metal ions, form the basis of stimulation responsive properties of DNA nanostructures.^[^ ^9^, ^19^, ^20^, ^21^, ^22^, ^23^ ^]^

**Scheme 1:**
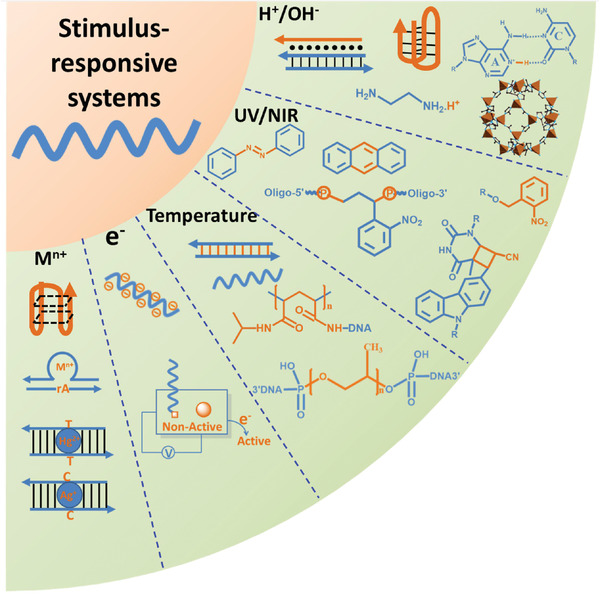
Stimuli‐responsive units that depend on different exogenous stimuli in DNA nanotechnology.

In addition to DNA, various organic molecules and polymers, as well as chemical bonds and chemical reactions, also process stimulation responsive properties. As for organic molecules, including the protonation and deprotonation of ethylenediamine (EN)^[^ ^24^, ^25^ ^]^ or polyaniline,^[^ ^26^ ^]^ photoisomerization of azobenzene,^[^ ^27^, ^28^, ^29^, ^30^ ^]^ and photochromism of anthracene,^[^ ^31^ ^]^ while for organic polymers, mainly thermal shrinkage of poly(propylene oxide) (PPO) and poly‐*N*‐Isopropylacrylamide (pNIPAM).^[^ ^32^, ^33^, ^34^ ^]^ Moreover, the disulfide bond can be photolyzed under pyrene, *o*‐nitrobenzyl (ONB) and its derivatives, (6‐nitropiperony) loxymethylene (NPOM), (2‐nitrovaleryl) oxymethyl (NVOM) can be used as photosensitive cages of DNA structures,^[^ ^35^, ^36^, ^37^, ^38^, ^39^ ^]^ molecular malachite green carbinol base (MGCB) can act as a light‐induced hydroxide ion emitter,^[^ ^40^ ^]^ the coordination bonds between zinc and imidazole can break under the regulation of pH values,^[^ ^41^ ^]^ all of these involve the breaking of chemical bonds. Examples of stimuli‐responsive chemical reactions include the [2+2]‐photocycloaddition reaction between the 3‐cyanovinyl carbazole DNA nucleoside and the thymine base diagonally opposite,^[^ ^42^, ^43^, ^44^ ^]^ the electron‐transfer reaction of metal ion,^[^ ^45^ ^]^ and the electron‐transfer reaction of thiol‐gold bond.^[^ ^45^ ^]^ These stimuli‐responsive systems can also realize the dynamic regulation of DNA nanostructures by connecting, modifying, embedding or mixing with DNA (**Scheme** **1**).^[^ ^46^, ^47^ ^]^

The development of DNA nanotechnology has brought unprecedented brilliance to the precise control of DNA nanostructures. However, the demand for the improvement of the control ability of conformation, function, and biocompatibility of DNA nanostructures has always existed. Therefore, a rich variety of DNA assembly‐based stimuli‐responsive systems have been developed through the combination of stimuli‐responsive units and DNA nanotechnology. These systems not only maintain the accuracy, stability, and codeability of DNA itself, but also have the rapid, remote, reversible dynamic regulation capability provided by stimuli‐responsive units. These features have stimulated much interest in developing novel dynamic DNA nanostructures to overcome the challenges in static DNA nanotechnology.

Over the past two decades, the application of DNA assembly‐based stimuli‐responsive systems has led to unprecedented dynamic DNA nanotechnology, which is mainly manifested in the nanofabrication of DNA architectures and biomedical applications. The DNA assembly‐based stimuli‐responsive systems can be controlled by exogenous stimuli, allowing for the design of DNA architectures with controllable conformational changes, as thus have become a powerful tool for the construction of dynamic DNA architectures. Towards biomedical needs and microenvironment in vivo, these dynamic DNA architectures can be widely used in biosensing, bioimaging, and biotherapy, which have greatly promoted the development of biomedicine.

In this review, we specifically summarize DNA assembly‐based stimuli‐responsive systems and their advances in DNA architectures and biomedical applications. We first elucidate the response mechanisms of stimuli‐responsive units that depend on different exogenous stimuli (DNA strand, pH, light, temperature, electricity, metal ions, etc.). Subsequently, we introduce the application of DNA assembly‐based stimuli‐responsive systems in nanofabrication according to the composition and structure of DNA architectures. The inevitable evolution of the aforementioned nanofabrications in biomedical applications as biological research progresses was also discussed. Finally, we summarize the driving effect of DNA assembly‐based stimuli‐responsive system on DNA nanotechnology and the possible future development direction, opportunities, and challenges. We hope this review will provide new insights into DNA nanotechnology.

## 2 DNA Assembly‐Based Stimuli‐Responsive DNA Systems

### 2.1 DNA Strand‐Responsive Systems

The precise Watson–Crick base pairing of the DNA enables the DNA strand itself to act as a stimuli‐responsive unit. Through additional DNA strands, or even a series of DNA reactions, such as strand‐displacement reactions (SDR), catalytic hairpin assembly (CHA) reactions, and hybridization chain reactions (HCR),^[^ ^48^ ^]^ the precise regulation of DNA assembly‐based systems can be realized. For example, DNA strand can be used as a “catalyst” for the “click reaction” to realize the ligation and assembly of DNA‐modified nanomaterials,^[^ ^49^, ^50^ ^]^ and SDR can be used as a tool to regulate DNA assembly^[^ ^51^ ^]^ or enzymatic biocatalytic cascades,^[^ ^52^ ^]^ which plays a great role in nanofabrication of DNA architectures.

### 2.2 pH‐Responsive Systems

At present, the pH‐responsive system in DNA nanotechnology can be mainly divided into four categories, i.e., the i‐motif structure, the triplex structure, the A+–C pairs, the protonated and deprotonated of the organic molecules, and the coordinate bond. The i‐motif, triplex, and A+–C pairs are DNA structures that have pH‐responsive properties themselves, whereas organic molecules and coordination bonds dynamically regulate the DNA structure by connecting, modifying, embedding or mixing with DNA or influence on related cofactors. These five types of stimulation responsive units are discussed in detail below.

#### 2.2.1 i‐Motif

**Figure 1:**
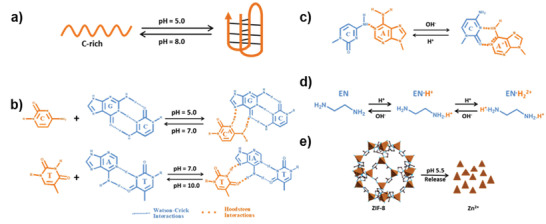
Representative examples of the pH‐responsive DNA structures a–c), organic molecule d), and coordination bonds e). a) Working cycle of an i‐motif structure. Reproduced with permission.^[^ ^63^ ^]^ Copyright 2003, Wiley‐VCH. b) Reversible formation of two pH‐responsive three‐base complex. Reproduced with permission.^[^ ^20^ ^]^ Copyright 2017, Wiley‐VCH. c) The pH‐responsive A^+^•C wobble base pairs. d) Schematic diagram of pH‐responsive ethylenediamine (EN). Reproduced with permission.^[^ ^24^ ^]^ Copyright 2018, Wiley‐VCH. e) The pH‐triggered release of Zn^2+^‐ion cofactors form ZIF‐8. Reproduced with permission.^[^ ^41^ ^]^ Copyright 2019, Wiley‐VCH.

Since 1962, scientists have observed cytosine‐protonated cytosine (C·C^+^) base pairs in acetyl cytosine crystals,^[^ ^53^ ^]^ RNA,^[^ ^54^, ^55^ ^]^ and DNA^[^ ^56^ ^]^ successively. In 1993, Gehring et al. reported that the cytosine‐rich DNA oligomers were a four‐strand complex at acidic pH, in which two base‐paired parallel‐stranded duplexes were intimately associated, with their base pairs fully intercalated. They designated this complex as the “i‐motif.”^[^ ^8^ ^]^ Since then, the study of i‐motif has been carried out rapidly,^[^ ^57^, ^58^, ^59^, ^60^, ^61^, ^62^ ^]^ and the self‐assembly of i‐motif at the acidic pH value (≈5.0) is gradually developed to a universal tool for designing the switchable DNA structures (**Figure** **1**a).^[^ ^9^ ^]^ One of the representative works was the proton–DNA nanomachine designed by Liu et al.,^[^ ^63^ ^]^ which places the nanomachine in a reversible cycle of pH = 5.0 and pH = 8.0, resulting in a cyclic switchable generation of i‐motif and duplex DNA structures. From a chemist's point of view, the i‐motif structure has a pH stimuli‐responsive property, so that it can be used not only as a biological target for chemical intervention but also for the synthesis of the intelligent functional nanostructures.^[^ ^21^, ^64^ ^]^ Up to date, i‐motif sequences have been widely used in the construction of nanomachines, nanopores, logic systems, pH sensors, DNA origami, hydrogel, and functional nanostructures combined with other nanomaterials,^[^ ^9^, ^21^, ^46^, ^64^, ^65^ ^]^ showing great application potential.

#### 2.2.2 Triplex

In nearly 50 years of research, different triplex structures were found between homopurine–homopyrimidine duplexes and single‐strand oligonucleotides,^[^ ^66^, ^67^ ^]^ including parallel and antiparallel triplex structures.^[^ ^10^, ^68^ ^]^ Among them, the third strand of parallel triplex structure forms a typical triplet state by Hoogsteen base pairing: T–A•T and C–G•C^+^ bind to high purine chain.^[^ ^20^, ^69^ ^]^ The low pH value is beneficial to the protonation of cytidine and as a consequence the stability of the parallel triplex will be greatly improved. Moreover, the C–G•C^+^ tends to form under acidic pH and T–A•T tends to form under neutral pH (Figure 1b).^[^ ^69^, ^70^, ^71^, ^72^, ^73^, ^74^ ^]^ Therefore, their pH responsive range can be regulated by the reasonable configuration of two Hoogsteen base pairing, so as to realize the control and reconfiguration of the DNA structure.

#### 2.2.3 A^+^•C Wobble

A^+^•C wobble is a common protonated base pairing in RNA and DNA,^[^ ^75^, ^76^ ^]^ which is flexible and not as rigid as a normal base pairing. It could swing in a certain pH range (Figure 1c),^[^ ^77^ ^]^ and the most predominant of which are the protonated wobble and the neutral reverse wobble.

#### 2.2.4 Organic Molecules

Organic molecules have diverse structures and adjustable properties, making them promising candidates for interacting with and regulating DNA structures. Many nitrogen‐containing organic molecules can be protonized and deprotonized under the regulation of solution pH.^[^ ^78^, ^79^ ^]^ Mao's group creatively applied the protonation properties of ethylenediamine (EN) to the dynamic DNA assembly, in which the protonated EN can promote the self‐assembly of negatively charged DNA backbones by overcoming the electrostatic repulsions (Figure 1d).^[^ ^24^ ^]^ Because there is no dependency on the DNA sequence, this method is expected to be a universal pH‐stimuli‐responsive strategy.

#### 2.2.5 Coordination Bonds

Both the metal ions and protons belong to Lewis acids, and they can compete for binding to the ligands. Therefore, coordination bonds formed by metal ions and ligands are pH‐responsive,^[^ ^80^, ^81^ ^]^ providing the theoretical basis for the application of pH‐based coordination bonds in DNA nanotechnology. Most recently, Wang's group developed a versatile Ce6‐DNAzyme@ZIF‐8 nanoplatform, in which the DNAzyme was encapsulated in the pH‐responsive ZIF‐8 nanoparticles (with coordination bonds formed by zinc ions and imidazoles), resulting a self‐sufficient DNA enzyme nanosystem (Figure 1e).^[^ ^41^ ^]^ This attempt fully demonstrates the application potential of pH‐responsive coordination systems in the field of DNA nanotechnology.

### 2.3 Photoresponsive Systems

The wavelength and intensity of light can be regulated accurately, and the process of light activation is usually completely reversible.^[^ ^47^, ^82^, ^83^ ^]^ These unique properties make light an ideal external control element in dynamic DNA nanotechnology. The most widely used photoresponsive system in dynamic DNA technology is “photosensitizer.”

According to the mechanism or wavelength responsiveness of photosensitizer, it can be divided into four categories: 1) conformational change photosensitizer, whose conformation can change reversibly when exposed to light (e. g., azobenzene and its derivatives, *n*‐methyl‐arylazole, stilbene, alkene‐based rotary molecular motors);^[^ ^27^, ^28^, ^84^, ^85^, ^86^, ^87^, ^88^, ^89^ ^]^ 2) reactive photosensitizer, which can stimulate the reaction of itself or other structure after being irradiated by light (e. g., anthracene, malachite green carbinol base (MGCB), photocleavable bonds, photocycloaddition reaction, aromatic hydrocarbons, chlorin e6 (Ce6));^[^ ^31^, ^40^, ^44^, ^90^, ^91^, ^92^, ^93^ ^]^ 3) photochemical cage, which is a photodissociation group that can be dissociated by light (e. g., *o*‐nitrobenzyl (ONB) and its derivatives, (6‐nitropiperony) loxymethylene (NPOM), (2‐nitrovaleryl) oxymethyl (NVOM));^[^ ^35^, ^36^, ^37^, ^38^, ^39^, ^94^, ^95^ ^]^ 4) photosensitive nanoparticles, which can initiate photothermal reaction (e. g., gold nanoparticles (AuNPs)),^[^ ^96^, ^97^, ^98^, ^99^, ^100^ ^]^ or photoconversion reaction (e. g., upconversion nanoparticles (UCNPs)).^[^ ^101^, ^102^, ^103^, ^104^ ^]^

#### 2.3.1 Conformational Change Photosensitizer

**Figure 2:**
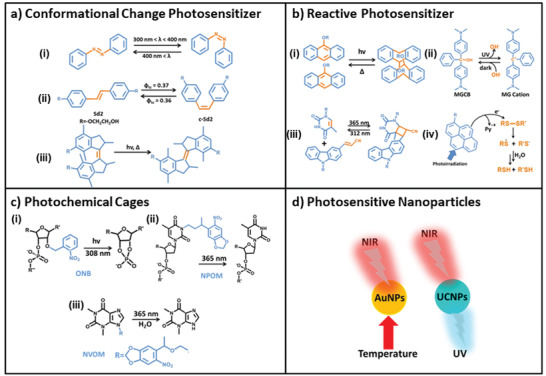
Representative examples of photoresponsive units. Working principles of conformational change photosensitizers a), reactive photosensitizers b), photochemical cages c), and photosensitive nanoparticles d). a) Schematic representation of the photoregulated azobenzene i), stilbene diether ii), and alkene‐based rotary molecular iii). i) Reproduced with permission.^[^ ^28^ ^]^ Copyright 2007, Nature Publishing Group. ii) Reproduced with permission.^[^ ^89^ ^]^ Copyright 2002, American Chemical Society. iii) Reproduced with permission.^[^ ^84^ ^]^ Copyright 2018, American Chemical Society. b) Diagrammatic representation of anthracene photochromism involving a [4*π* + 4*π*] photocycloaddition reaction, forming a head‐to‐tail isomer i), MGCB photoresponsive emission of OH^−^ ii), photoregulated hybridization through [2+2]‐photocycloaddition reaction iii), and pyrene‐assisted photolysis of disulfide iv). i) Reproduced with permission.^[^ ^31^ ^]^ Copyright 2019, Royal Society of Chemistry. ii) Reproduced with permission.^[^ ^40^ ^]^ Copyright 2007, Wiley‐VCH. iii) Reproduced with permission.^[^ ^42^ ^]^ Copyright 2015, American Chemical Society. iv) Reproduced with permission.^[^ ^91^ ^]^ Copyright 2012, Wiley‐VCH. c) The mechanism of photochemical cages of ONB, NPOM, and NVOM (caging groups were shown in blue). i,ii) Reproduced with permission.^[^ ^38^ ^]^ Copyright 2010, American Chemical Society. iii) Reproduced with permission.^[^ ^39^ ^]^ Copyright 2006, Elsevier. d) Photothermal reaction of AuNPs and photoconversion reaction of UCNPs.

Conformational change photosensitizer is the most widely studied photosensitizer. They switch between *cis*‐ and *trans*‐ isomers, or interconvert between closed and open forms (**Figure** **2**a).^[^ ^47^ ^]^ Taking azobenzene, the most widely studied photosensitizer, as an example, its *trans* conformation is more stable than the *cis*‐conformation. Under ultraviolet light, the *trans*‐isomer can be converted to *cis*‐isomer to achieve the regulation of DNA structures through modification on DNA.^[^ ^28^ ^]^ In addition, stilbene,^[^ ^85^, ^89^ ^]^ alkene‐based rotary molecular motor^[^ ^84^ ^]^ can realize the dynamic regulation of DNA nanostructures through conformational changes.

#### 2.3.2 Reactive Photosensitizer

Beyond the conformational change, some of the photosensitizers can react under the irradiation of light, stimulate the occurrence of other reactions (Figure 2b), which can be directly or indirectly applied to DNA nanotechnology.^[^ ^31^, ^40^, ^44^, ^90^, ^91^ ^]^ First, we present a few representative examples of photosensitizers that photoinduce their own reactions. Anthracene can dimerize by the [4*π* + 4*π*] photocycloaddition reaction under light irradiation, and modification of the adaptor by anthracene groups can realize the optical reversible control of the catalytic process;^[^ ^31^ ^]^ MGCB can emit hydroxide ions under the initiation of light to regulate the pH of the solution, thus achieving reversible control of the pH‐responsive DNA nanostructure.^[^ ^40^ ^]^ Photocleavable bonds can be cleaved by light, and the modification of DNA by photocleavable bonds can control the reaction of DNA.^[^ ^90^ ^]^ There are also photosensitizers that, when exposed to light, can stimulate reactions of other structures or between them and other structures. For example, 3‐cyanovinyl carbazole DNA nucleoside can react with a thymine base diagonally opposite via a [2+2]‐photocycloaddition reaction to form a cyclobutane;^[^ ^42^, ^43^, ^44^ ^]^ pyrene molecules can efficiently facilitate the photolysis of disulfide bonds within artificial nucleic acid backbones;^[^ ^91^, ^105^ ^]^ Ce6 can mediate the conversion of oxygen to reactive oxygen species (ROS),^[^ ^92^, ^93^ ^]^ and it has many advantages such as large absorption coefficient in infrared region and small toxicity, so it is widely used in DNA‐based gene silencing photodynamic therapy (PDT).^[^ ^41^, ^106^, ^107^ ^]^

#### 2.3.3 Photochemical Cages

Photosensitive cage is a special type of photosensitizer, which is generally used in DNA nanotechnology to temporarily mask Watson–Crick base and inhibit the catalytic ability of ribozyme.^[^ ^35^, ^36^, ^37^, ^38^, ^39^, ^94^, ^95^ ^]^ It can dissociate under light to restore the catalytic performance of ribozyme. At present, various photosensitive cages have been reported (Figure 2c), including ONB and its derivatives, NPOM, and NVOM, which realized the regulation of ribozyme catalytic ability through their photodissociation performance.^[^ ^38^, ^39^, ^94^ ^]^

#### 2.3.4 Photosensitive Nanoparticles

Because the near‐infrared (NIR) light has deep tissue penetration capability, the photosensitive nanoparticles with the response to the NIR light show a unique advantage in the field of DNA‐based biological diagnosis and treatment. Here we introduce two kinds of NIR light‐responsive nanoparticles widely used in DNA nanotechnology: AuNPs and UCNPs (Figure 2d). AuNPs have a strong absorption band in the NIR light region, and the absorbed photon energy can be converted into heat with high efficiency under the irradiation of NIR light. This heat can cause changes in the surrounding DNA systems with thermal response. Therefore, AuNPs are considered as an excellent noninvasive adjuvant for photothermal therapy (PTT). Unlike the photothermal conversion properties of AuNPs, UCNPs can continuously absorb low‐energy NIR light to emit high‐energy ultraviolet (UV) light or visible light. Based on the above characteristics, the photosensitive nanoparticles have realized the nucleic acid‐based therapy and bioimaging by means of connection with the functionalized DNA.^[^ ^37^, ^108^ ^]^

### 2.4 Temperature‐Responsive Systems

The melting temperature (*T*_m_) is an important parameter of DNA, which is the temperature when the duplex structure of 50% DNA molecule is opened. There is a positive relationship between the *T*_m_ value of double‐stranded DNA (dsDNA) and the content of G–C, that the *T*_m_ value of dsDNA generally increases with the amounts of G–C base pairs, which is the theoretical basis of the thermal dynamics of DNA.^[^ ^109^, ^110^ ^]^ The temperature‐responsive system plays an irreplaceable role in DNA nanotechnology. Studies have shown that both heating^[^ ^111^, ^112^, ^113^, ^114^, ^115^ ^]^ and cooling^[^ ^116^, ^117^, ^118^ ^]^ can bring fascinating changes to DNA. In addition to the temperature responsiveness of DNA itself, some organic polymers with temperature‐responsive properties, such as poly(propylene oxide) (PPO), and poly(*N*‐isopropylacrylamide) (pNIPAM)^[^ ^119^ ^]^ have also been used in DNA nanotechnology.^[^ ^32^, ^33^, ^34^, ^119^ ^]^

#### 2.4.1 DNA Helix

**Figure 3:**
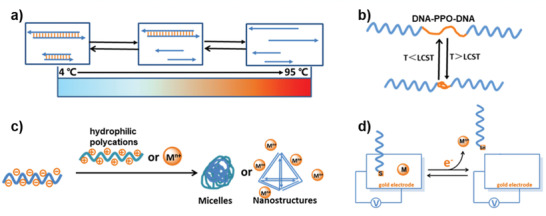
Schematic illustration of temperature and electrical‐responsive systems. a) The temperature‐responsive behavior of DNA helix. b) The thermal responsiveness of DNA–polymer–DNA complex. Reproduced with permission.^[^ ^34^ ^]^ Copyright 2018, Wiley‐VCH. c) Fabrication of DNA micelles and nanostructures with electrostatic responsiveness based on the electrical properties of DNA. d) The electron‐transfer reaction‐based molecule release from the electrode surface. Reproduced with permission.^[^ ^45^ ^]^ Copyright 2016, Royal Society of Chemistry.

Because of the thermal stability of DNA, different DNA helix structures can be opened to different extents as the temperature increases, so that the behavior of DNA helix can be controlled by temperature (**Figure** **3**a).^[^ ^112^, ^113^, ^114^, ^115^ ^]^ The different temperature responsiveness of dsDNA molecules of different lengths can also be used in the assembly and disassembly of DNA nanostructures.^[^ ^111^ ^]^ Furthermore, using a combination of thermoswitches of different stabilities or a mix of stabilizers of various strengths, extended thermometers that respond linearly up to 50 °C in temperature range can be designed.^[^ ^112^ ^]^

In addition to heating, the effect of cooling on DNA has gradually attracted research interest. Hervé’s group has shown that the cooling rate has a decisive effect on the conformation of specific DNA;^[^ ^116^ ^]^ Liu's group demonstrated the effects of freezing on the stretching and alignment of single‐stranded DNA (ssDNA)^[^ ^117^ ^]^ and hybridization of very short DNA.^[^ ^118^ ^]^

#### 2.4.2 Temperature‐Responsive of Organic Polymers

The thermal responsiveness of organic polymers is closely related to their low critical solution temperature (LCST). When the temperature is higher than LCST, the polymers are hydrophobic and in contraction state. When the temperature is lower than LCST, the polymer will be converted to hydrophilic, restoring relaxation.^[^ ^32^, ^33^, ^34^, ^119^ ^]^ Thermally responsive polymer–DNA connections can therefore be used for reversible assembly of DNA‐based nanostructures. Willner's group modified the DNA with the temperature‐responsive pNIPAM to synthesize a reversibly temperature‐responsive DNA hydrogel.^[^ ^32^ ^]^ Liu's group implemented thermal‐triggered frame‐guided assembly of vesicles^[^ ^33^ ^]^ and temperature‐responsive DNA hydrogels^[^ ^34^ ^]^ by modifying the DNA with the thermal‐responsive PPO (Figure 3b).

### 2.5 Electrical‐Responsive Systems

#### 2.5.1 Electrical Property of DNA

DNA are large, hydrophilic, and negatively charged molecules,^[^ ^120^, ^121^, ^122^ ^]^ so they do not easily break through the electrostatic barrier and cross the cell membrane with the anionic nature. Therefore, the design of DNA architectures with electrostatic responsiveness based on the electrical properties of DNA has aroused the interest of scientists (Figure 3c).^[^ ^123^, ^124^, ^125^, ^126^, ^127^, ^128^ ^]^ Tirrell's group combined biocompatible, neutral, hydrophilic polymers, such as polyethylene glycol (PEG), with hydrophilic polycations such as polyethylenimine and polylysine (pLys), and mixed them with anion single‐ or double‐stranded DNA to produce polyelectrolyte composite micells (PCMs) having different shapes and sizes. Polyelectrolyte complexes neutralize the charge of DNA and protect them from degradation, while the corona provides additional shielding and reduces immunogenicity.^[^ ^125^ ^]^ Fan's group improved the cellular uptake efficiency of DNA through the “corner attack” of tetrahedral DNA nanostructures (TDNs) on cell membranes.^[^ ^126^ ^]^ In addition, they found that the shape reconstruction of DNA nanotubes could be driven by charge neutralization through changing the type and concentration of ions or through chemical modification of the DNA backbone.^[^ ^127^ ^]^ More recently, Lam's group constructed a Mg^2+^/EDTA‐controlled molecular switch which shows instant and reversible structural conversions at room temperature by the shielding effect of Mg^2+^ on electrostatic DNA duplexes.^[^ ^129^ ^]^ All of these studies point the way to research the electrical properties of DNA itself.

#### 2.5.2 Electron‐Transfer Reactions

Electron‐transfer reaction is also an effective means of controlling DNA directly or indirectly. Voltage can be applied to the surface of the electrode to control electron transfer across the electrode‐solution interphase through electronic input, facilitating redox reactions in a highly controlled manner, leading to the release of molecular inputs that regulate the behavior of DNA.^[^ ^45^, ^130^, ^131^, ^132^ ^]^ Hyun's group constructed an electrochemical reversible DNA switch by the redox of lead ions (Pb^2+^) on the electrode,^[^ ^130^ ^]^ while Ricci's group constructed three kinds of DNA‐based nanoswitches by the redox of the mercury ion (Hg^2+^), the thiol‐gold bond and the copper ion (Cu^2+^), respectively (Figure 3d).^[^ ^45^ ^]^ The electron‐transfer reaction provides a general method for electrochemical regulation of the DNA.

### 2.6 Metal Ion‐Responsive Systems

Due to the metal ion‐dependence of DNAzymes such as peroxidase‐mimicking DNAzyme and RNA‐cleaving DNAzyme, and ionic bridging complex such as T–Hg^2+^–T and C–Ag^+^–C, metal ions are widely used in DNA assembly‐based stimuli‐responsive systems.

#### 2.6.1 DNAzyme

**Figure 4:**
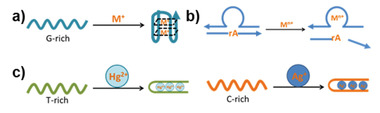
Schematic illustration of representative working mechanisms of metal ion‐responsive systems. The mechanism of peroxidase‐mimicking DNAzyme a), RNA‐cleaving DNAzyme b), and ionic bridging complex c). a,b) Reproduced with permission.^[^ ^133^ ^]^ Copyright 2009, American Chemical Society. c) Reproduced with permission.^[^ ^134^ ^]^ Copyright 1992, Nature Publishing Group.

DNAzyme is a kind of catalytic nucleic acid isolated by in vitro screening technique. Over the past two decades, DNAzymes, particularly horseradish peroxidase‐mimicking DNAzyme and RNA‐cleaving DNAzyme, have been used in various fields of DNA nanotechnology. G‐rich sequences can form G‐quadruplex in the presence of potassium ions (**Figure** **4**a),^[^ ^6^, ^133^, ^135^, ^136^, ^137^, ^138^, ^139^, ^140^ ^]^ while the embedding of hemin gives it the catalytic properties of peroxidase‐mimicking^[^ ^48^, ^141^, ^142^, ^143^, ^144^, ^145^, ^146^, ^147^ ^]^ and the potential to act as the electron transfer (ET) receptor.^[^ ^148^, ^149^, ^150^, ^151^ ^]^ RNA‐cleaving DNAzyme catalyze nucleic acid substrate cleaving by using target metal ions as cofactors (Figure 4b).^[^ ^134^ ^]^ The discovery of different metal ions (e.g., Cu^2+^, Mg^2+^, Pb^2+^, Zn^2+^, Mn^2+^)‐dependent DNAzymes, make RNA‐cleaving DNAzyme a powerful tool for detecting or regulating metal ions.^[^ ^134^, ^152^, ^153^ ^]^ These two DNAzymes, alone or combined, are widely used in DNA nanotechnology.^[^ ^9^, ^139^, ^152^, ^154^, ^155^, ^156^, ^157^, ^158^, ^159^, ^160^, ^161^, ^162^, ^163^ ^]^ Horseradish peroxidase‐mimicking DNAzyme are usually the colorimetric output units,^[^ ^48^, ^141^, ^142^, ^143^, ^144^, ^145^, ^146^, ^147^ ^]^ while the RNA‐cleaving DNAzyme is usually used as sensing units of metal ions.^[^ ^134^, ^152^, ^153^ ^]^ Combining these two DNAzymes effectively, it can form dual DNAzyme systems which can realize the functions of sensing and signal output simultaneously.^[^ ^156^, ^161^, ^163^ ^]^

#### 2.6.2 Ion‐Bridging Complex

In 2003, scientists discovered that the nonspecific binding of mercury cations (Hg^2+^) and silver cations (Ag^+^) in the DNA double strand unexpectedly and significantly stabilized the naturally occurring mismatch base pairs (T–T, C–C) to form ion‐bridging complexes: T–Hg^2+^–T and C–Ag^+^–C (Figure 4c).^[^ ^13^ ^]^ Since then, a variety of stimuli‐responsive systems based on ion‐bridging complex have been established.^[^ ^14^, ^164^, ^165^, ^166^ ^]^ The ion‐bridging complex is not only limited to the construction of simple Hg^2+^/Ag^+^ sensors,^[^ ^14^, ^167^, ^168^, ^169^ ^]^ but also plays an important role in logic gate,^[^ ^170^ ^]^ DNA nanostructure,^[^ ^171^ ^]^ and DNA machines.^[^ ^172^, ^173^ ^]^

### 2.7 Multiple Stimuli Cooperative Responsive Systems

With the gradual maturity of stimuli‐responsive system in DNA nanotechnology, multiple stimuli cooperative responsive systems have been developed by the combination of two or more stimuli‐responsive units. Such as the light/electricity/heat and pH co‐controlled i‐motif structure,^[^ ^40^, ^174^, ^175^ ^]^ as well as the pH/light/heat and metal ions coordinated control of catalytic performance of DNAzyme.^[^ ^138^, ^176^, ^177^ ^]^ Based on the photothermal effect of Au–Ag nanorods (Au–Ag NRs) and temperature‐response of dsDNA, Tan's group constructed the near‐infrared light‐responsive core–shell nanogels,^[^ ^178^ ^]^ while Lu's group constructed the light/metal ion coordinated controlled DNAzyme–gold nanoshells by further introduction of DNAzyme.^[^ ^108^ ^]^

Notably, aptamers are another type of functional nucleic acids in addition to DNAzyme, which are generally single‐stranded DNA or RNA molecules that can specifically bind to their target molecules, including metal ions, small molecules, and protein.^[^ ^179^ ^]^ They can fold into different secondary and tertiary structures after the binding. Based on its specific recognition performance and ability to regulate DNA conformation, recent research efforts have emerged to produce bifunctional biomolecular structures (aptazymes) by combining DNAzymes with aptamers, in which the aptamer act as binding sites and the DNAzyme act as catalytic site.^[^ ^180^ ^]^ The aptazyme has multiple response sites, so it becomes an ideal platform for the construction of multiple stimuli‐responsive system. For example, aptazyme with enhanced catalytic activities has been constructed while the dopamine aptamer act as a binding site, and the DNAzyme act as the catalytic unit.^[^ ^181^ ^]^ The amplification detection of target protein could be achieved by the aptamer based DNAzyme motor. For example, in the presence of target protein and Mg^2+^, the binding of protein triggers sequential cleavage of DNAzyme substrates on AuNPs, releasing fluorescence signals.^[^ ^182^ ^]^ Compared to systems only responsive to single stimuli, multiple stimuli cooperative responsive systems may have greater application potential in biomedical research fields such as biosensing, bioimaging, drug delivery, and theranostics, in which complex cellular environment are involved.

## 3 Applications of Stimuli‐Responsive DNA Systems

### 3.1 Nanofabrication of DNA Architectures

The single Watson–Crick base pairing in the nucleic acid limits the flexibility of the nucleic acid assembly. Thus, the introduction of stimuli‐responsive elements to enrich the assembly of nucleic acids has attracted the interest of scientists. DNA assembly‐based stimuli‐responsive systems show outstanding performance in nanofabrication of various DNA architectures, including both pure and hybrid DNA architectures, ranging from simple 1D to complex 2D or even 3D DNA architectures. Moreover, inspired by complex mechanical functions in nature and the dynamic interactions in organisms, DNA assembly‐based stimuli‐responsive systems have also shown application potential in the construction of DNA nanomachines mimicking mechanical functions and constitutional dynamic networks mimicking natural networks.

#### 3.1.1 Fabrication of Pure DNA Nanostructures

##### 1D DNA Architectures

**Figure 5:**
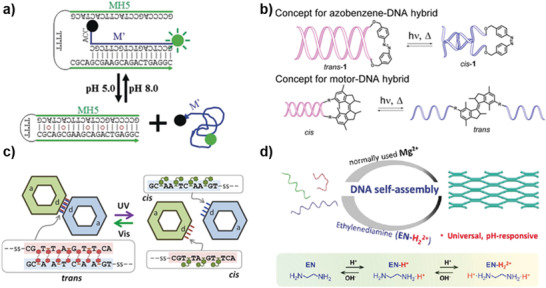
Nanofabrication of one‐ and 2D DNA architectures based on DNA assembly‐based stimuli‐responsive systems. a) Rational design of pH‐responsive DNA motifs based on the A^+^–C pairs. Reproduced with permission.^[^ ^76^ ^]^ Copyright 2019, Wiley‐VCH. b) Schematic overview of photoswitchable DNA hairpins based on alkene‐based molecular motor molecular motors. Reproduced with permission.^[^ ^84^ ^]^ Copyright 2018, American Chemical Society. c) Reversible photoregulation for the formation and dissociation of hexagonal dimer under different irradiation conditions. Reproduced with permission.^[^ ^189^ ^]^ Copyright 2012, American Chemical Society. d) pH‐controlled reversible assembly and disassembly of 2D DNA arrays in an EN/TA buffer. Reproduced with permission.^[^ ^24^ ^]^ Copyright 2018, Wily‐VCH.

Assembly in 1D DNA architectures mainly refers to the simple assembly of nucleic acid chains. Over the past decade, scientists have used pH,^[^ ^76^ ^]^ light,^[^ ^28^, ^29^, ^82^, ^84^, ^183^, ^184^, ^185^, ^186^, ^187^ ^]^ temperature,^[^ ^117^, ^118^, ^188^ ^]^ and metal ions^[^ ^166^ ^]^ to regulate the reversible assembly of nucleic acid chains. For example, Fu et al. reported a pH‐responsive DNA motifs with general sequence compatibility through the introduction of A^+^•C wobbles, in which they could regulate the bonding strength between the DNA strands and realize the reversible assembly and dissociation of DNA duplex (**Figure** **5**a).^[^ ^76^ ^]^ The reversible regulation of nucleic acid chain assembly by light is mainly achieved by photoisomerization of conformational change photosensitizer, and the reversible regulation of DNA and PNA assembly has been implemented.^[^ ^28^, ^29^, ^82^, ^183^, ^184^, ^185^, ^187^ ^]^ By fusing an azobenzene with a natural T base, Tan's group designed the photosensitive artificial base (zT) to realize the light‐controlled reversible assembly of DNA strands,^[^ ^183^ ^]^ while Lubbe et al. reported the photoswitching of DNA hybridization using the alkene‐based molecular motor (Figure 5b).^[^ ^84^ ^]^ More recently, Liu's group proved that low temperature has an effect on the secondary structure and binding ability of the DNA strands, which should be attributed to the temperature responsiveness of DNA itself.^[^ ^117^, ^118^ ^]^ Li's group demonstrated that silver and mercury ions could control the direction of DNA assembly by forming ion bridging complex.^[^ ^166^ ^]^ Both of them provide novel methods for the assembly of DNA strands.

##### 2D DNA Architectures

Introduction of DNA assembly‐based stimuli‐responsive systems increases the complexity and functionality of simple DNA architectures, making them ideal units for advanced structural reassembly, gradually driving the nanofabrication of 2D DNA architectures, including DNA origami,^[^ ^189^, ^191^, ^192^, ^193^ ^]^ arrays,^[^ ^24^ ^]^ and tiles.^[^ ^194^ ^]^

Using the triplex structures or DNAzymes as a junction unit for DNA origami, Willner's group designed the pH and metal ion‐stimulated reversible dimer and trimer assembly systems of origami.^[^ ^191^, ^192^, ^193^ ^]^ Taking the reversible reconstruction of DNA origami dimers based on two kinds of DNAzymes as an example, they introduce both RNA‐cleaving DNAzymes and G‐quadruplex DNAzymes into the intermediate linker of DNA origami. The addition of K^+^‐ion induces the formation of G‐quadruplexes, which leads to the reconstruction of origami dimers and this reconstruction can achieve a reversible process in the presence of 18‐crown‐6‐ether.^[^ ^193^ ^]^ Sugiyama's group obtained the predesigned multiorientational patterns of the hexagonal DNA origami, and realized reversible assembly and disassembly of DNA origami through the introduction of photosensitive azobenzene (Figure 5c).^[^ ^189^ ^]^ Mao's group achieved reversible self‐assembly of pH‐responsive 2D DNA arrays through the introduction of protonated organic molecules (EN) (Figure 5d),^[^ ^24^ ^]^ while Racci's group designed the pH‐controlled self‐assembly of 2D DNA tiles by introducing triplex into the strand displacement circuit.^[^ ^194^ ^]^

##### 3D DNA Architectures

Introduction of DNA assembly‐based stimuli‐responsive systems also drive the nanofabrication of 3D DNA architectures, such as DNA tubes,^[^ ^131^, ^195^, ^199^ ^]^ multiscaffold DNA origami objects,^[^ ^196^, ^200^ ^]^ crystals,^[^ ^201^, ^202^, ^203^ ^]^ hydrogels,^[^ ^73^, ^111^, ^171^, ^197^, ^204^, ^205^, ^206^ ^]^ tetrahedron,^[^ ^190^ ^]^ dendrimer,^[^ ^207^ ^]^ raft,^[^ ^208^ ^]^ and nanopore.^[^ ^198^ ^]^

**Figure 6:**
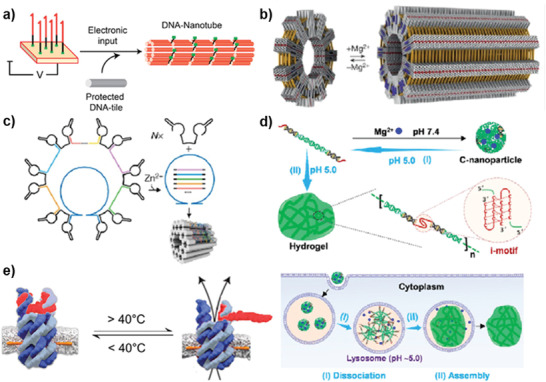
Nanofabrication of 3D DNA architectures based on DNA assembly‐based stimuli‐responsive systems. a) Remote electronic control of DNA nanotube self‐assembly. Reproduced with permission.^[^ ^131^ ^]^ Copyright 2018, American Chemical Society. b) Assembly of self‐limiting ring oligomers into tubes by increasing the ionic strength of the solution. Reproduced with permission.^[^ ^195^ ^]^ Copyright 2017, Nature Publishing Group. c) Schematic representation of biotechnological production of DNA origami by the DNAzymes. Reproduced with permission.^[^ ^196^ ^]^ Copyright 2017, Nature Publishing Group. d) Schematic illustration of topological transformation from nanoparticles to organelle‐like hydrogel by pH, and endosomal escape of the organelle‐like hydrogel architecture. Reproduced with permission.^[^ ^197^ ^]^ Copyright 2020, Wiley‐VCH. e) Reversibly opens and closes of temperature‐gated DNA nanopore. Reproduced with permission.^[^ ^198^ ^]^ Copyright 2019, American Chemical Society.

For example, Ricci's group have introduced pH and electrical response systems into the reversible assembly of DNA nanotubes.^[^ ^131^, ^199^ ^]^ For pH‐driven reversible self‐assembly of DNA nanotube, a regulating strand and a triplex‐based pH‐sensing strand ware used to regulate the assembly and dissociation of DNA tiles.^[^ ^199^ ^]^ The electronic‐driven reversible self‐assembly of DNA nanotube was achieved by remotely controlling the desorption of the DNA input strands through electron transfer reaction via the cathodic potential (**Figure** **6**a).^[^ ^131^ ^]^ By increasing ionic strength to overcome static electricity between electronegative DNA blocks, Dietz's group realized the assembly of gigadalton‐scale DNA tubes (Figure 6b).^[^ ^195^ ^]^ In addition, they reported that the RNA‐cleaving DNAzyme could cleave single‐stranded precursor DNA generated by bacteriophages to short strand for DNA origami assembly, realizing mass production of DNA origami (Figure 6c).^[^ ^196^ ^]^

The stimuli‐response system has also been introduced into the assembly of DNA crystals, and Seeman's group has done remarkable work in this regard.^[^ ^201^, ^202^, ^203^ ^]^ For instance, they realized fabrication of macroscopic 3D crystals based on the threefold rotationally symmetric tensegrity triangle that can be functionalized by a triplex‐forming oligonucleotide (TFO) on each of its helical edges,^[^ ^201^ ^]^ which may offer applications such as the organization of nanoelectronics, the direction of biological cascades, and the structure determination of periodically positioned molecules by X‐ray diffraction.^[^ ^201^ ^]^ Also, they organized an organic semiconductor molecule in 3D DNA crystals by binding pH‐responsive octaniline molecules into DNA scaffolds. They obtained four different states of octaniline in a single crystal based on the redox and protonation chemistry of polyaniline,^[^ ^203^ ^]^ which is of great guiding significance for the construction of more complex electronic systems.

Stimuli‐responsive structures have been prominent in the reversible assembly of pure DNA hydrogels.^[^ ^73^, ^111^, ^171^, ^204^, ^205^, ^206^ ^]^ For example, through the introduction of i‐motif, the pH‐responsive reversible pure DNA hydrogels were designed by Liu's group.^[^ ^204^, ^206^ ^]^ They also designed a pure DNA hydrogel with temperature‐responsive properties through different thermal stability of different lengths of DNA.^[^ ^111^ ^]^ Willner's group reported Ag^+^‐stimulated pure DNA hydrogel with the C–Ag^+^–C ion‐bridging complexes,^[^ ^171^ ^]^ while Murata's group obtained a pure DNA hydrogel with photoresponsive property by the assembly and dissociation of X‐shaped DNA motif (X‐motif) through the introduction of [2+2]‐photocycloaddition reaction.^[^ ^205^ ^]^ More recently, Yang's group constructed organelle‐like pure hydrogels in living cells through the shielding of negative DNA charges by metal cations and the responsiveness of i‐motif structures to pH (Figure 6d), which was achieved by the lysosomal acidic microenvironment induced topological transformation from nanoparticles to organelle‐like hydrogel architecture.^[^ ^197^ ^]^ This study took pure DNA hydrogels a step forward as nano‐biomedicines.

What's more, the pH‐responsive DNA tetrahedron^[^ ^190^ ^]^ and size‐tunable DNA dendrimers^[^ ^207^ ^]^ were constructed by the introduction of triplex structure and i‐motif structures respectively. The thermal stability properties of DNA was used to design temperature‐responsive nanopore (Figure 6e).^[^ ^198^ ^]^ Based on the [2+2]‐photocycloaddition reaction, Seeman's group designed an exponential‐grown DNA origami rafts which could produce more than 7 million copies in 24 cycles under temperature control and ultraviolet illumination. Also, they demonstrated the selectivity of DNA origami rafts growth through the introduction of a triplex structure.^[^ ^208^ ^]^

#### 3.1.2 Hybrid DNA Architectures

Owing to the unique features of DNA, such as strong modifiability and precise programmable, special consideration is given to using DNA to guide the nanofabrication of hybrid DNA architectures, including DNA‐guided nanoparticle assemblies,^[^ ^42^, ^209^, ^210^, ^211^, ^213^, ^214^, ^215^, ^216^, ^217^, ^218^, ^219^ ^]^ DNA–protein nanomaterials,^[^ ^220^ ^] [^ ^221^ ^]^ polyelectrolyte complex micelles (PCMs),^[^ ^125^ ^]^ microcapsules,^[^ ^212^ ^]^ vesicles,^[^ ^33^ ^]^ as well as hybrid DNA hydrogels.^[^ ^32^, ^70^, ^71^, ^72^, ^222^, ^223^, ^224^, ^225^, ^226^, ^227^, ^228^, ^229^, ^230^, ^231^ ^]^

**Figure 7:**
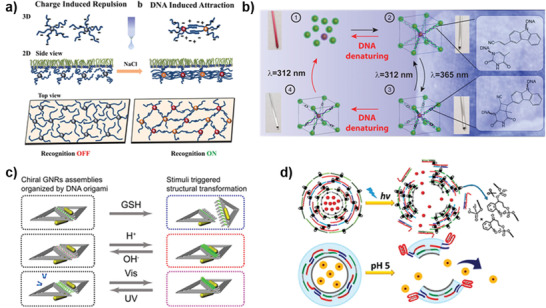
Nanofabrication of hybrid DNA architectures based on DNA assembly‐based stimuli‐responsive systems. a) Schematic illustration of the assembly of DNA‐functionalized nanoparticles at positively charged interfaces. Reproduced with permission.^[^ ^209^ ^]^ Copyright 2014, American Chemical Society. b) Reversible photochemical ligation of nanoparticle superlattices through the introduction of [2+2]‐photocycloaddition reaction. Reproduced with permission.^[^ ^210^ ^]^ Copyright 2014, American Chemical Society. c) Schematic illustration of the pH‐responsive and photoresponse GNR plasmonic nanostructures. Reproduced with permission.^[^ ^211^ ^]^ Copyright 2017, American Chemical Society. d) Schematic presentation of the photocleavable/pH‐responsive DNA microcapsules. Reproduced with permission.^[^ ^212^ ^]^ Copyright 2016, American Chemical Society.

A variety of stimuli‐responsive strategies have been established for reversible assembly of nanoparticles and quantum dots (QDs).^[^ ^42^, ^209^, ^210^, ^211^, ^213^, ^214^, ^215^, ^216^, ^217^, ^218^, ^219^ ^]^ For example, based on the thermal stability of complementary DNA molecules attached to the surface of the AuNPs, Gang's group reported the formation of thermal‐responsive 3D crystal of AuNPs in 2008.^[^ ^219^ ^]^ Later, they reported the construction of 2D nanoparticle membrane at liquid interfaces by modulating the electrostatic repulsion of AuNPs modified with electronegativity DNA on the surface (**Figure** **7**a).^[^ ^209^ ^]^ More recently, they obtained the light‐induced reversible AuNP superlattices through the introduction of [2+2]‐photocycloaddition reaction in DNA connector (Figure 7b).^[^ ^210^ ^]^ The peroxidase‐mimicking DNAzyme (G‐quadruplex) was used by Willner's group to construct K^+^‐responsive reversible assembly of QDs.^[^ ^214^ ^]^ With the participation of i‐motif structure or conformational change photosensitizer‐azobenzene moieties, Liu's group reported a stimuli‐responsive plasmonic nanosystem based on DNA origami organized gold nanorods (GNRs),^[^ ^211^ ^]^ whose nanoarchitectures can be reversibly regulated by the stimulation of pH change or photoirradiation respectively (Figure 7c).

The introduction of stimuli‐responsive systems in the assembly of DNA and other substances such as proteins, metal–organic frameworks, calcium carbonate and organic polymers could develop a series of reversible response architectures.^[^ ^33^, ^41^, ^125^, ^186^, ^212^, ^220^, ^221^, ^232^, ^233^, ^234^, ^235^ ^]^ For example, Uyeda's group reported the photoregulated reversible assembly of azobenzene‐modified DNA‐templated protein arrays in 2010.^[^ ^220^ ^]^ Pei's group reported a 2D “DNA−protein” nanomaterials regulated by G‐quadruplex and i‐motif.^[^ ^221^ ^]^ Tirrell's group synthesized polyelectrolyte complex micelles (PCMs) with different morphology dependent on DNA electrical properties by DNA oligonucleotides of varied length and hybridization state and poly(l)lysine‐poly(ethylene glycol) block copolymers with varying block lengths.^[^ ^125^ ^]^ Willner's group developed microcapsules that respond to pH or light based on the layer‐by‐layer deposition of sequence‐specific nucleic acids, which was linked with PC bond or i‐motif sequences, on poly(allylamine hydrochloride)‐functionalized CaCO_3_ core microparticles (Figure 7d).^[^ ^212^ ^]^ Liu's group reported a thermally triggered frame‐guided assembly (FGA) strategy for the preparation of vesicles, in which the thermally responsive poly(propylene oxide) (PPO) was used to make the leading hydrophobic groups (LHGs) thermally responsive.^[^ ^33^ ^]^ Stimuli‐responsive DNA system as a platform for complex structural construction of biological and organic materials is rapidly developing.

The hybrid DNA hydrogel is also a very important member of hybrid DNA architectures. Development of stimuli‐responsive hybrid hydrogels greatly reduces the cost of pure DNA hydrogels and provides a good platform for expanding bioanalysis and biomedical applications of DNA assembly‐based stimuli‐responsive systems. Willner's group have developed various hybrid DNA hydrogels that are responsive to multiple sources of stimuli.^[^ ^32^, ^70^, ^71^, ^72^, ^222^, ^223^, ^224^, ^225^, ^226^, ^227^, ^228^ ^]^ For example, they revealed a stimuli‐triggered reversible shape regulatory by adding pH‐responsive triplex structure and different stimuli‐responsive units (pNIPAM, i‐motif and K^+^) to the two‐layered hydrogels for assembly of asymmetrically mixed DNA‐based hydrogels.^[^ ^228^ ^]^ Recently, they designed DNA hybrid hydrogels that respond to light or K^+^ through the introduction of photosensitizer dithienylethene (DTE) and G‐quadruplexes.^[^ ^227^ ^]^ By introducing the i‐motif structure, Liu's group reported the pH‐responsive DNA–SWNT hybrid hydrogel.^[^ ^229^ ^]^ Later, they reported a supramolecular polypeptide–DNA hydrogel, which could achieve the control of the mechanical properties of the hydrogel by regulating the thermal stability of the DNA linker or the pH‐dependent conformation of the polypeptide backbone.^[^ ^230^ ^]^ Recently, they synthesized thermally responsive hybrid DNA hydrogel by uniformly inserting a thermally responsive polymer (PPO), into a rigid 3D DNA network.^[^ ^34^ ^]^

#### 3.1.3 DNA Nanomachines

Besides simple assembly, using DNA as a material to respond to external stimuli and switches on machine functions is an exciting opportunity to develop DNA nanomachines.^[^ ^19^, ^23^, ^240^, ^241^, ^242^, ^243^ ^]^ Hence, it is not all too surprising, scientists have introduced stimuli‐responsive DNA structures into the design of various DNA nanomachines, including nanoswitches,^[^ ^9^, ^45^, ^112^, ^130^, ^174^, ^244^, ^245^, ^246^, ^247^, ^248^, ^249^, ^250^ ^]^ walkers,^[^ ^91^, ^172^, ^251^, ^252^, ^253^, ^254^, ^255^ ^]^ tweezers,^[^ ^143^, ^256^, ^257^, ^258^, ^259^, ^260^, ^261^ ^]^ rotors,^[^ ^173^, ^237^, ^259^, ^262^, ^263^, ^264^ ^]^ and conformational nanomachines.^[^ ^63^, ^265^, ^266^, ^267^, ^268^, ^269^, ^270^, ^271^, ^272^ ^]^

A DNA switch is a supramolecular nucleic acid assembly that undergoes cyclic, switchable, transitions between two distinct states in the presence of appropriate triggers.^[^ ^9^ ^]^ Therefore, the stimuli‐responsive system has undoubtedly become an ideal element for building DNA switches.^[^ ^45^, ^112^, ^130^, ^174^, ^244^, ^245^, ^246^, ^247^, ^248^, ^249^, ^250^, ^273^ ^]^ For example, Yu's group reported a robust electronic switch made of immobilized ion‐responsive peroxidase‐mimicking DNAzyme, which could achieve switching operation under the action of K^+^ and crown‐6.^[^ ^245^ ^]^ Fan's group reported the i‐motif switch realized by electrochemically controlling the pH of the solution.^[^ ^174^ ^]^ Racci's group reported triplex switches whose pH‐dependent properties could be regulated by altering the relative content of the T–A•T/C–G•C^+^ triplets.^[^ ^248^ ^]^ Later they engineered the triplex structure into an octahedral DNA nanostructure for a reversible opening/closing switching.^[^ ^249^ ^]^ They also promote electron transfer reaction by applying different voltage potentials on the surface of the electrode, releasing molecular input (metal ions or specific DNA sequences) from the electrode surface and triggering DNA‐based nanoswitches or nanodevices.^[^ ^45^ ^]^

DNA nano tweezers or scissors that respond to external stimuli have been widely developed as a class of nanoswitches with special configuration. For example, Asanuma's group constructed light‐responsive DNA tweezers through the introduction of photosensitive azobenzene.^[^ ^257^ ^]^ Willner's group built multiple pH‐responsive DNA tweezers by introducing i‐motif structure.^[^ ^143^, ^258^, ^259^ ^]^

**Figure 8:**
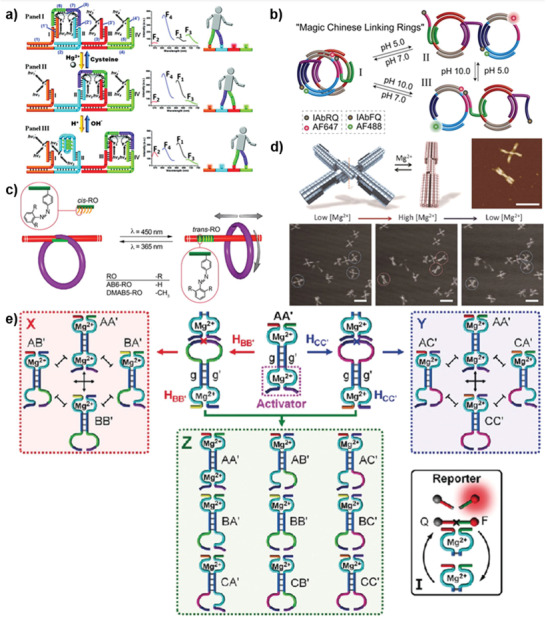
Nanofabrication of DNA nanomachines based on DNA assembly‐based stimuli‐responsive systems. a) Bipedal walker activated by Hg^2+^/cysteine and H^+^/OH^−^ inputs. Reproduced with permission.^[^ ^172^ ^]^ Copyright 2011, American Chemical Society. b) Programmed pH‐stimulated dissociation and association of a two circular DNA construct crosslinked by two pH‐responsive locks. Reproduced with permission.^[^ ^236^ ^]^ Copyright 2016, American Chemical Society. c) Reversible toehold‐release‐ODN induced pseudorotaxane–rotaxane switch. Reproduced with permission.^[^ ^237^ ^]^ Copyright 2012, American Chemical Society. d) DNA origami nanoscissor (NS) switch showing open/closed dynamic motion responsive to the salt concentration. Reproduced with permission.^[^ ^238^ ^]^ Copyright 2017, Wiley‐VCH. e) Emergence of DNA‐based constitutional dynamic networks, for example, simple [2 × 2] networks and a complex [3 × 3] network. Reproduced with permission.^[^ ^239^ ^]^ Copyright 2019, Wiley‐VCH.

Notably, Mao's group reported a DNA nanodevice that autonomously and processively moves along a DNA track based on the based on the programmed cleavage of the RNA‐cleaving DNAzyme under the stimulation of metal ions in 2005,^[^ ^251^ ^]^ which provided a basis for application of DNA assembly‐based stimuli‐responsive systems in DNA walker. After that, a bipedal walker and stepper was designed by Willner's group through the introduction of ionic bridging complexes and i‐motif structures (**Figure** **8**a).^[^ ^172^ ^]^ Tan's group designed DNA walkers that responded to light by using the photosensitive azobenzene^[^ ^253^ ^]^ or pyrene molecules^[^ ^91^, ^255^ ^]^ as the photosensitive units. DNA walkers have driven the DNA nanotechnology to be more intelligent and bio‐enabled.

DNA assembly‐based stimuli‐responsive systems are also introduced into interconnected circular DNA structures.^[^ ^236^, ^274^ ^]^ By combining the two two‐ring nanostructure together through two different pH‐responsive triplex structures (T–A•T and C–G•C^+^), Willner's group obtained interlocked circular DNA dimers with bidirectional reversible assembly and dissociation properties (Figure 8b).^[^ ^236^ ^]^ By systematically studying the folding of various intramolecular and intermolecular i‐motif DNAs, Li and Famulok achieved programmable functionalization of DNA circles.^[^ ^274^ ^]^

More importantly, reconfiguration of the interlocked structures allowed the operation of DNA machines such as spring, rotors, and rotaxane.^[^ ^173^, ^237^, ^259^, ^262^, ^263^, ^264^ ^]^ For example, Ren's group designed a pH‐responsive DNA nanospring using the i‐motif structure as the linker for multiple DNA rings.^[^ ^264^ ^]^ Famulok's group realized the phototriggered reversible motion of the DNA ring on the rotaxane axis by the conformational change photosensitizer‐2′, 6′‐dimethylazobenzene functionalized release oligodeoxynucleotides (ODNs) (Figure 8c).^[^ ^237^ ^]^ Also, they designed chain structures controlled by pH‐responsive i‐motif structures.^[^ ^263^ ^]^ Two‐station^[^ ^259^ ^]^ or three‐station^[^ ^173^ ^]^ DNA catenane rotary motors have also been developed by introducing i‐motif structures and ion‐bridging complexes (T–Hg^2+^–T) into the DNA rings, as well as the five‐ and seven‐ring catenanes.^[^ ^262^ ^]^

Meanwhile, the stimuli‐responsive system can induce conformational changes in a variety of DNA nanostructures, forming nanomachines based on conformational changes, including 3D framed nucleic acid (FNA) nanomachines,^[^ ^269^, ^270^ ^]^ and DNA origami nanomachines.^[^ ^238^, ^268^, ^275^, ^276^ ^]^ For example, Fan's group constructed DNA nanomachines through the i‐motif conformation regulation in DNA tetrahedron.^[^ ^270^ ^]^ Through the charge shielding effect of metal cations on DNA or the introduction of the photosensitizer (azobenzene or [2+2]‐cycloaddition reaction), Dietz's group designed a series of DNA origami nanomachines with response to metal ions (Figure 8d)^[^ ^238^, ^268^, ^276^ ^]^ or light,^[^ ^238^, ^275^ ^]^ which achieve a higher structural level of dynamic regulation of the DNA nanostructures.

#### 3.1.4 Constitutional Dynamic Networks

The stimuli‐triggered constitutional dynamic network is a recently rapidly developing topic, which is aiming to assemble “artificial cells” by stimuli‐responsive DNA modules to mimic intracellular dynamic interactions between DNA, RNA, and proteins.^[^ ^277^ ^]^ The DNA‐based stimuli‐responsive system provides various communication pathways for constitutional dynamic networks. At present, different stimuli‐responsive DNA systems have been used to control the constitutional dynamic network. These includes metal ions,^[^ ^239^, ^278^ ^]^ light,^[^ ^279^ ^]^ and additional DNA strands.^[^ ^51^, ^280^, ^281^, ^282^, ^283^, ^284^, ^285^, ^286^, ^287^, ^288^ ^]^ For example, DNA modules functionalized with Mg^2+^ ion‐dependent DNAzyme activator can be used to construct a versatile rewiring mechanism that leads to the emergence of DNA‐based constitutional dynamic networks.^[^ ^239^ ^]^ As shown in Figure 8e, AA′ activate the intact hairpin *H*_BB′_ to form the [2 × 2] constitutional dynamic network X, while subjecting AA′ to an intact hairpin *H*_CC′_ leads to the generation of [2 × 2] constitutional dynamic network Y. Combine the two, AA′ activate the *H*_BB′_ and *H*_CC′_ results in the [3 × 3] constitutional dynamic network Z. It would be challenging to realize the vision to design “artificial cell.” However, in view of the promising development of DNA‐based constitutional dynamic networks, it offers exciting opportunities to scale up systems chemistry.

### 3.2 Biomedical Applications

Stimuli‐response systems have greatly promoted the rapid development of DNA nanotechnology in biomedical applications, such as biosensing, bioimaging, and therapy.

#### 3.2.1 Biosensors

Sensors are devices that respond to physical or chemical stimuli and produce detectable signals, while the stimuli‐responsive system in DNA nanotechnology is characterized by a specific, fast, and reversible response to physical or chemical stimuli, so it is an ideal “element” in sensor construction.^[^ ^32^, ^47^, ^130^, ^289^ ^]^ Up to date, DNA assembly‐based stimuli‐responsive systems have been widely used for the biochemical analysis of metal ions, nucleic acids, proteins, small molecules, bacteria, as well as pH values, UV light, and temperatures.^[^ ^20^, ^155^, ^290^, ^291^, ^292^, ^293^ ^]^

##### Metal Ions Sensors

**Figure 9:**
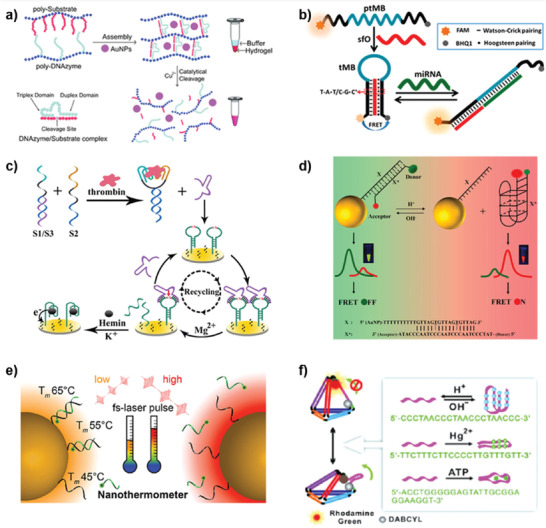
Biosensors based on DNA assembly‐based stimuli‐responsive systems. a) The working principle of copper ion responsive DNAzyme crosslinked hydrogel. Reproduced with permission.^[^ ^294^ ^]^ Copyright 2011, Royal Society of Chemistry. b) Schematic illustration of the analysis of miRNA using the classical tMB. Reproduced with permission.^[^ ^74^ ^]^ Copyright 2018, American Chemical Society. c) Schematic presentation of the proximity binding and metal‐ion dependent DNAzyme‐based recycling amplification for electrochemical assay of thrombin. Reproduced with permission.^[^ ^295^ ^]^ Copyright 2016, American Chemical Society. d) Working principle of the dual‐fluorophore‐labeled i‐motif nanoprobe. Reproduced with permission.^[^ ^296^ ^]^ Copyright 2015, American Chemical Society. e) Schematic showing the AuNP‐dsDNA system, local heating due to laser‐induced photothermal event and subsequent DNA release. Reproduced with permission.^[^ ^297^ ^]^ Copyright 2020, American Chemical Society. f) Scheme of DNA tetrahedra that are reconfigurable by adapting dynamic sequences in one arm (i‐motif, T‐Hg^2+^‐T). Reproduced with permission.^[^ ^170^ ^]^ Copyright 2012, Wiley‐VCH.

Various metal ion sensors based on DNAzymes and ion‐bridging complexes have been developed.^[^ ^155^, ^158^, ^159^ ^]^ The catalytic activity of RNA‐cleaving DNAzymes usually requires metal ions (e.g., Pb^2+^, Zn^2+^, Mg^2+^, Mn^2+^, Ca^2+^, Cu^2+^, UO^2+^, and Na^+^) cofactors, so the RNA‐cleaving DNAzyme system is an ideal system for metal ion detection.^[^ ^158^ ^]^ In 2000, Lu's group developed a lead ion sensor based on RNA‐cleaving DNAzymes, which realized the highly sensitive and specific detection of Pb^2+^.^[^ ^298^ ^]^ Since then, a series of metal ion sensors based on RNA‐cleaving DNAzymes have flourished,^[^ ^35^, ^152^, ^294^, ^298^, ^299^, ^300^, ^301^, ^302^, ^303^, ^304^, ^305^, ^306^, ^307^, ^308^ ^]^ including the colorimetric sensor of RNA‐cleaving DNAzymes binding gold nanoparticles (AuNPs)^[^ ^152^, ^302^, ^307^, ^308^ ^]^ or DNA hydrogels.^[^ ^294^, ^300^, ^301^ ^]^ For example, using DNAzyme‐directed assembly of AuNPs, Lu's group first constructed a colorimetric biosensor for Pb^2+^.^[^ ^152^ ^]^ Pb^2+^‐dependent DNAzyme catalyzes the hydrolysis of its substrate and bring the aggregated AuNPs return to a dispersed state in the presence of Pb^2+^, resulting a color change of the sensor solution from blue to red. Yang's group constructed a series hydrogels for visual detection of metal ions.^[^ ^294^, ^300^, ^301^ ^]^ In these systems, the enzyme strand and substrate strand of RNA‐cleaving DNAzyme were incorporated into liner polyacrylamide respectively, and the 3D hydrogel would form by the crosslink of the enzyme strand and substrate strand to trap AuNPs in the hydrogel pores. The introduction of target metal ions would induce the cleavage of the substrate strands, following by the dissolution of hydrogel and the release of AuNPs, which could be visually measured (**Figure** **9**a).^[^ ^294^ ^]^ Notably, the K^+^ could significantly stabilize the G‐quadruplex/hemin DNAzyme with horseradish peroxide‐mimic properties,^[^ ^6^, ^7^ ^]^ which could be used as a platform for K^+^ detection. For example, Dong's group has constructed a colorimetric method for K^+^ detection by using the G‐quadruplex DNA aptamer as the sensing element.^[^ ^309^ ^]^

The specific recognition properties of ion‐bridging complexes for Hg^2+^ and Ag^+^ make them an important recognition element of the metal ion sensors, and has been widely used to construct fluorescent, colorimetric, chemiluminescent, and electrochemical sensors for Hg^2+^ and Ag^+^.^[^ ^14^, ^168^, ^310^, ^311^, ^312^, ^313^, ^314^, ^315^ ^]^ Based on the formation of mercury‐mediated base pairs (T–Hg^2+^–T), Ono's group fabricated an oligodeoxyribonucleotide (ODN)‐based fluorescence sensing system that selectively detects Pb^2+^ by employing two T‐rich sequences and a linker.^[^ ^14^ ^]^ Through the HCR initiated by Ag^+^‐bridging complex, Liu et al. realized the sensitive electrochemical impedance spectroscopy (EIS) detection of Ag^+^.^[^ ^312^ ^]^

In addition, the cooperative detection of metal ions has been realized by employing DNAzymes and bridging complexes as the stimuli‐responsive units.^[^ ^139^, ^167^, ^168^ ^]^ For example, highly sensitive fluorescence detection of Hg^2+^ has been enabled through Hg^2+^ triggered activation of RNA‐cleaving DNAzyme.^[^ ^167^ ^]^ Colorimetric detection of Hg^2+^ was realized through inhibition of G‐quadruplex/hemin DNAzyme formation by Hg^2+^/G‐quadruplex complexes.^[^ ^139^ ^]^

##### Nucleic Acids Sensors

A series of nucleic acid sensors based on DNAzymes have been developed by promoting or inhibiting the formation of DNAzymes with nucleic acid.^[^ ^150^, ^162^, ^316^, ^317^, ^318^, ^319^, ^320^ ^]^ For example, the fluorescence detection of target DNA was achieved through the activation of Mg^2+^‐dependent DNAzyme systems by target DNA.^[^ ^316^ ^]^ Zhang's group achieved fluorescence detection of target RNA through the inhibiting effect of target RNA on the formation of G‐quadruplex/hemin DNAzyme and the photoinduced electron transfer (PET) process with oligonucleotide‐templated silver nanoclusters (DNA‐AgNCs).^[^ ^150^ ^]^

In addition to DNAzymes, azobenzene and triplex structures are also used in nucleic acid detection.^[^ ^74^, ^256^ ^]^ Yuan's group designed a 3D DNA nanostructure based on the azobenzene‐functionalized DNA nippers, which could realize the photoresponsive capture and release of the microRNAs (miRNAs).^[^ ^256^ ^]^ The electrochemiluminescence (ECL) detection of miRNAs could be realized by the modification of ECL emitters Ru(bpy)_2_^2+^ and the quencher Alexa Fluor (AF). Yang's group has achieved the detection of miRNAs by manipulating the strength of the stem‐binding in the triplex molecular beacon (tMB) using pH values (Figure 9b).^[^ ^74^ ^]^

##### Protein and Small Molecule Sensors

The aptamers of a variety of proteins and small molecules have been used in sensing systems combined with stimuli‐responsive DNA nanotechnology.^[^ ^150^, ^182^, ^295^, ^321^, ^322^, ^323^, ^324^, ^325^, ^326^ ^]^ For example, a pH‐based ATP fluorescence sensing system was realized by regulation of the distance between the two segments of aptamer by the i‐motif structure.^[^ ^326^ ^]^ Xiang's group developed a thrombin detection strategy by integrating proximity binding‐induced strand displacement and metal ion‐dependent DNAzyme recycling amplification (Figure 9c).^[^ ^295^ ^]^

Human telomerase is a ribonucleoprotein that can add hexamer DNA repeat (TTAGGG)*_n_* to the end of telomere during DNA replication. Using telomerase to extend the substrate strand of RNA‐cleaving DNAzyme, Tan's group realized the detection of telomerase.^[^ ^327^ ^]^ Telomerase detection methods in living cells based on similar mechanisms have also been reported.^[^ ^328^ ^]^

pH Sensors: As the pH‐responsive DNA nanostructure, i‐motif and triplex structures have been used to construct pH sensors.^[^ ^296^, ^329^, ^330^, ^331^, ^332^ ^]^ Molecular sensors that can respond to 0.2 pH units and above were obtained by reasonably regulating the structure of i‐motif and adding allosteric control elements.^[^ ^330^ ^]^ What's more, the ratiometric fluorescent nanoprobe for sensing pH values in living cells was also designed and fabricated.^[^ ^296^ ^]^ The dual‐fluorophore‐labeled i‐motif sequences immobilized on the AuNP surface would fold into a C‐quadruplex structure and detach from the surface of AuNPs under acidic conditions.^[^ ^296^ ^]^ Accompanied by the occurrence of FRET between the two fluorophore groups, fluorescence detection of the pH response was realized in living cells.^[^ ^296^ ^]^ Recently, Ricci's group reported a triplex structure‐modified urease‐powered micromotors,^[^ ^332^ ^]^ which could monitor the pH values of their surrounding microenvironment during self‐propulsion. Temperature sensors: A series of DNA nanothermometers have been developed based on the thermodynamic properties of DNA itself.^[^ ^112^, ^113^, ^114^, ^115^, ^297^, ^333^ ^]^ For example, Yang's group reported an intracellular thermometer based on the thermal stability of non‐natural l‐DNA molecular beacon (l‐MB).^[^ ^115^ ^]^ Using structural modifications or inexpensive DNA stabilizers, Vallée‐Bélisle's group extended the linear response temperature range, and got programmable quantitative DNA nanothermometers with a linear range up to 50 °C.^[^ ^112^ ^]^ Díaz and Oh's group constructed a quantitative nanothermometer based on the femtosecond (fs) laser pulsed excitation of plasmonic nanoparticle (NP)−double‐stranded DNA (dsDNA) conjugates, in which the localized DNA denaturation rate could be modulated by varying pulse energy fluence, DNA melting temperature, and surrounding bath temperature (Figure 9e).^[^ ^297^ ^]^ Famulok's group developed a thermal nanosensors by the assembly of two interlocked dsDNA rings, which contain a group of thermal‐responsive DNA joints whose temperature response range can be easily tuned by changing the length or the sequence of the hybridized region in its structure.^[^ ^333^ ^]^

##### Logic Gates

Multiple sensing system is an excellent candidate for building logic circuits. A variety of stimuli sources, such as proton,^[^ ^170^, ^258^, ^263^, ^272^ ^]^ metal ions,^[^ ^7^, ^170^, ^313^, ^314^, ^334^, ^335^, ^336^, ^337^ ^]^ photosensitive molecules,^[^ ^88^ ^]^ can be used as input signals to design multiple logic gates.^[^ ^160^, ^338^ ^]^ For example, with the employment of i‐motif structures, ion‐bridging complexes and aptamer, Fan's group realized the sensing of pH, Hg^2+^, and ATP by regulating the conformation of tetrahedral FNA (Figure 9f),^[^ ^313^ ^]^ which could perform a series of logic operations (INH, XOR, OR, and AND). Zhang's group constructed a complete set of two‐input logic gates (OR, AND, INHIBIT, XOR, NOR, NAND, and XNOR) based on the use of ion‐dependent DNAzymes as functional components and the respective metal ions as inputs.^[^ ^336^ ^]^ Famulok's group used two photosensitive molecules (DM‐AZO/AAP) to regulate the formation of split horseradish peroxidase‐mimicking DNAzyme to construct complex logical circuits using different wavelengths of light as inputs.^[^ ^88^ ^]^

#### 3.2.2 Bioimaging

**Figure 10:**
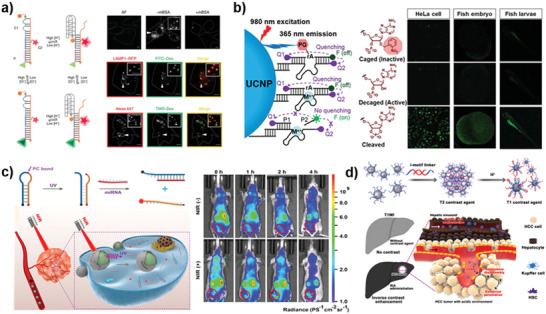
Bioimaging based on DNA assembly‐based stimuli‐responsive systems. a) Working principle and imaging of the two‐ion measurement fluorescent probe. Reproduced with permission.^[^ ^348^ ^]^ Copyright 2019, Nature Publishing Group. b) Schematic and imaging of the NIR metal ion sensor. A 2′‐nitrobenzyl photocage group (PG) is added to protect the ribonucleotide adenosine (rA) site in the substrate strand from being cleaved, which is photodissociated under 365 nm emission from UCNP. Reproduced with permission.^[^ ^37^ ^]^ Copyright 2018, American Chemical Society. c) Schematic and imaging of DNA nanodevice for NIR light activated miRNA sensing in vivo. Reproduced with permission.^[^ ^90^ ^]^ Copyright 2019, American Chemical Society. d) Schematic illustration of RIA for diagnosis of small HCC. Reproduced with permission.^[^ ^349^ ^]^ Copyright 2018, American Chemical Society.

Stable environmental and material levels in living cells are necessary to maintain the normal activity of living organisms. The DNA assembly‐based stimuli‐responsive systems exhibit the characteristics of rapid response and controllability, and thus have been applied to construct fluorescence imaging systems to monitor intracellular pH values,^[^ ^272^, ^296^, ^339^, ^340^, ^341^ ^]^ metal ion concentrations,^[^ ^35^, ^36^, ^37^, ^108^, ^177^, ^342^, ^343^, ^344^ ^]^ microRNA levels,^[^ ^90^, ^252^, ^345^, ^346^, ^347^ ^]^ and ATP concentrations.^[^ ^272^, ^341^ ^]^ For example, Krishnan's group successfully constructed a two‐ion measurement fluorescent probe through the introduction of pH sensitive i‐motif structures and chloride‐sensitive fluorescent groups to achieve simultaneous imaging of pH and chloride in the same lysosome, which could enable decoding the mechanistic underpinnings of lysosomal diseases, monitoring disease progression or evaluating therapeutic efficacy (**Figure** **10**a).^[^ ^348^ ^]^ Lu's group did a series of work on stimulating system‐based cellular imaging, enabling imaging of multiple metal ions^[^ ^35^, ^36^, ^37^, ^108^, ^343^, ^344^ ^]^ and microRNAs^[^ ^90^, ^252^ ^]^ in living cells. They achieved optical control over imaging of metal ions in living cells and in vivo by connecting DNAzymes protected by photosensitive cages on UCNPs (Figure 10b).^[^ ^37^ ^]^ Also, through the functionalization of UCNPs surfaces by PC bond‐modified nanodevices, they achieved the imaging of microRNAs in cells and tissues (Figure 10c).^[^ ^90^ ^]^ Li's group achieved imaging of endogenous ATP and H^+^ in lysosome through the reconstruction^[^ ^341^ ^]^ or conformation changes^[^ ^272^ ^]^ of i‐motif structure‐linked FNAs.

In addition to being applied to fluorescence imaging, the stimuli‐responsive system has also been applied to magnetic resonance (MR) imaging. For example, Ling's group constructed an i‐motif DNA‐assisted pH‐responsive iron oxide nanocluster assemblies (termed RIAs), which could response to the acidic pH of the tumor microenvironment, thus provide an inverse contrast enhancement effect to improve the distinction between normal liver and target hepatocellular carcinomas (HCCs) tissues (Figure 10d).^[^ ^349^ ^]^ Based on a HCR and DNAzyme biocatalysis, Wang's group constructed an autocatalytic DNAzyme (ACD) biocircuit sustained by a honeycomb MnO_2_ nanosponge (hMNS), which allows simultaneous fluorescence and MR imaging.^[^ ^350^ ^]^ The stimuli‐responsive imaging strategy greatly enriches the toolbox of techniques for bioanalysis in living systems, which is important in the diagnosis of human diseases, especially cancers.

#### 3.2.3 Cancer Therapy

At current stage, cancer therapy mainly includes chemotherapy, phototherapy, immunotherapy, and gene therapy.^[^ ^64^, ^179^ ^]^ DNA assembly‐based stimuli‐responsive systems play an important role in these individual methods of therapy or multi‐method synergistic therapy.

In chemotherapy, biological release and transport of drugs is a critical issue. The DNA assembly‐based stimuli‐responsive systems can be used as the gating of DNA system,^[^ ^249^, ^266^, ^339^, ^351^, ^352^, ^353^ ^]^ such as nanochannels,^[^ ^354^, ^355^, ^356^ ^]^ metal–organic frameworks (MOFs)^[^ ^41^, ^357^ ^]^ and mesoporous SiO_2_ nanoparticles (MP SiO_2_ NPs),^[^ ^346^, ^358^, ^359^, ^360^, ^361^, ^362^, ^363^ ^]^ and also participate in the assembly and degradation of microcapsules,^[^ ^212^, ^232^, ^364^ ^]^ hydrogels,^[^ ^178^, ^222^, ^365^, ^366^, ^367^ ^]^ polymers,^[^ ^106^, ^368^, ^369^ ^]^ and nanosponges.^[^ ^22^, ^370^, ^371^ ^]^ Therefore, the DNA assembly‐based stimuli‐responsive systems can be used as a trigger to control the release of nucleic acids, small molecules and drugs,^[^ ^22^, ^371^, ^372^ ^]^ which has been widely employed as a common strategy to build drug release system. For example, by switching the conformation of azobenzene‐incorporated DNA using visible and ultraviolet light alternately, Jiang's group fabricated light‐controlled nanochannels to regulate ion^[^ ^354^ ^]^ or ATP^[^ ^355^ ^]^ transport. Willner's group used i‐motif structure as the gating of metal–organic frameworks (MOFs),^[^ ^357^ ^]^ together with RNA‐cleaving DNAzymes as the gating of MP SiO_2_ NPs, to achieve drug release regulated by metal ions.^[^ ^359^ ^]^ Based on the photothermal effect of Au–Ag nanorods (Au–Ag NRs), Tan's group constructed a near‐infrared light‐responsive drug delivery and release platform. The Au–Ag NRs leads to a rapid temperature increase in the Au–Ag NRs coated hydrogels, resulting in the rapid release of the drug loading with light‐controllability.^[^ ^178^ ^]^ They also synthesized *o*‐nitrobenzyl‐modified aptamer‐grafted hyperbranched polymers, in which the hydrophilic properties of the polymer is controlled by the redox reaction of *o*‐nitrobenzyl under UV light, triggering the dissociation of the polymer and the release of the drug.^[^ ^369^ ^]^

**Figure 11:**
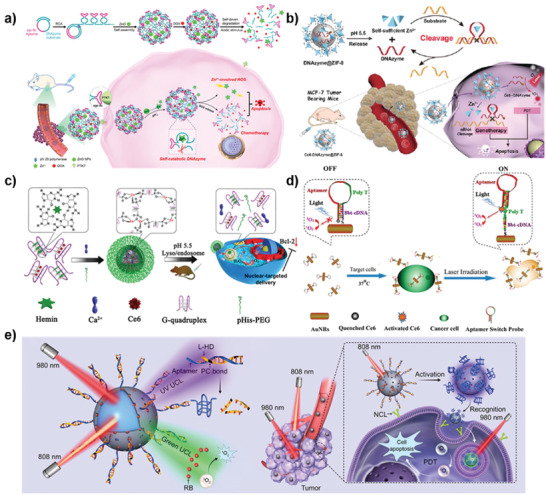
Cancer therapy based on DNA assembly‐based stimuli‐responsive systems. a) Schematic illustration of RCA‐based assembly and acid‐triggered disassembly of DNA nanosponge, as well as the intracellular ROS generation and drug release after specific cellular uptake. Reproduced with permission.^[^ ^370^ ^]^ Copyright 2019, American Chemical Society. b) The pH‐triggered DNAzyme@ZIF‐8 nanosystem for combined gene‐photodynamic therapy. Reproduced with permission.^[^ ^41^ ^]^ Copyright 2019, Wiley‐VCH. c) A schematic illustration for synthesis of Ca‐AS1411/Ce6/hemin@pHis‐PEG NCPs (CACH‐PEG). Reproduced with permission.^[^ ^106^ ^]^ Copyright 2018, American Chemical Society. d) Schematic representation of ASP‐photosensitizer‐AuNRs for PTT and PDT. Reproduced with permission.^[^ ^373^ ^]^ Copyright 2012, American Chemical Society. e) Schematic showing the orthogonal regulation of DNA nanodevice for programmed tumor cell recognition and treatment. Reproduced with permission.^[^ ^374^ ^]^ Copyright 2020, American Association for the Advancement of Science.

Combined with other nanomaterials, polymers, or DNA amplification techniques, delicate systems have been realized, capable of targeted drug delivery, remote pulsating drug release, and programmable drug release. For example, an intelligent and nonviolent self‐driven drug delivery platform was constructed by disassociating the pH‐responsive coordination bond in ZnO nanoparticles to provide supplement Zn^2+^ cofactors, which mediated the nonviolent DNAzyme‐catalyzed cleavage of DNA scaffolds for precise and efficient drug administrations with synergistically enhanced therapeutic performance (**Figure** **11**a).^[^ ^370^ ^]^

In addition to traditional chemotherapy, dynamic systems gradually play an important role in emerging cancer treatments, such as photothermal therapy (PTT),^[^ ^346^, ^373^ ^]^ PDT,^[^ ^41^, ^106^, ^107^, ^373^, ^374^ ^]^ and gene therapy.^[^ ^41^, ^106^, ^107^, ^346^, ^375^ ^]^ For example, Wang's group developed a Ce6‐DNAzyme@ZIF‐8 nanoplatform for imaging‐guided combined cancer gene‐photodynamic therapy, in which the ZIF‐8 would dissociate in the tumor acidic environment to release Ce6‐DNAzyme and the Zn^2+^ needed for the RNA‐cleaving DNAzyme. The Ce6 and RNA‐cleaving DNAzyme would trigger PDT and gene silencing, respectively, to achieve synergistic therapy (Figure 11b).^[^ ^41^ ^]^ They also constructed an intelligent multifunctional theranostic nanoplatform by encapsulating therapeutic DNAzyme prodrugs and MnO_2_ adjuvant into a thermo‐responsive nanocapsule, which not only realizes photo‐genetherapy, but also assists PDT, while providing strong T1 MR contrast agent for MR imaging.^[^ ^376^ ^]^ Chen's group developed a coordination‐driven self‐assembly method for the preparation of DNA‐based nanoscale coordination polymers (NCPs) by mixing Ca^2+^, AS1411 G‐quadruplex, and pHis‐PEG copolymer in aqueous solution. The Ce6 for PDT and the hemin for catalase‐mimicking DNAzyme could both insert to the AS1411 G‐quadruplex to obtain the Ca‐AS1411/Ce6/hemin@pHis‐PEG (CACH‐PEG) NCP nanostructure. The Ce6 could produce the required ROS in PDT, and the oxygen produced by catalase‐mimicking DNAzyme could improve the efficiency of PDT. Meanwhile, AS1411 G‐quadruplex could inhibit the expression of antiapoptotic protein B‐cell lymphoma 2 (Bcl‐2) and improve the apoptosis caused by PDT (Figure 11c).^[^ ^106^ ^]^ With the combination of photocaged DNA nanocombs and UCNP, Ju's group fabricated an microRNA amplifier through a microRNA triggered near‐infrared photoswitched cascade reaction. The precise PDT of early‐stage cancers can be achieved through the generation of ROS by the activation of photosensitizers.^[^ ^377^ ^]^ Tan's group linked the photosensitizer molecule chlorin e6 (Ce6) to the aptamer switch probe (ASP), which was modified on the surface of gold nanorods (AuNRs) for PTT, achieving synergistic therapeutic effect of cancer (Figure 11d).^[^ ^373^ ^]^ They also developed a Ce6‐DNAzyme‐MnO_2_ nanosystem for synergistic therapy with gene silence and PDT, in which MnO_2_ nanosheets were used as protective and activator of DNAzymes for gene silencing, and photosensitizer Ce6 for PDT.^[^ ^107^ ^]^ By combining an UV light‐activatable aptamer module and a photocleavable 2‐nitrobenzyl linker with UCNP that enables the operation of the nanodevice with deep tissue‐penetrable NIR light, a controllable therapeutic DNA devices with high spatial–temporal selectivity was developed recently, which could achieve the programmed tumor recognition and PDT (Figure 11e).

More recently, the research team of Tan co‐developed a programmable polymer library by including various stimuli‐responsive units in building blocks, and reasonably assembled a series of smart nanocarriers with hierarchical structures containing logic gates, which realized in vivo biological computing and multi‐therapy specific site delivery, providing a powerful tool for designing precise medicines personalized for the treatment of human diseases.^[^ ^378^ ^]^ This system successfully realized imaging‐release‐therapy integration, defining the direction for the development of stimuli‐responsive DNA nanotechnology in biomedical application.

## 4 Conclusions and Perspectives

Over the past decades, dynamic DNA nanotechnology regulated by DNA assembly‐based stimuli‐responsive systems emerges as the time requires, which enables accurate and predictable dynamic manipulation of DNA structures. The key to construct DNA assembly‐based stimuli‐responsive systems is to integrate DNA assemblies and stimuli‐responsive units together. Stimuli‐responsive units can be integrated with DNA assemblies in covalent and noncovalent manners. Once this integration is established, the fast, reversible, and remotely controllable properties of the stimuli‐responsive unit can be transferred to the DNA assembly, providing a rich “tool box” for the regulation of DNA nanostructures and functions.

Along with the rapid development of DNA nanotechnology and the increasing enrichment of stimuli‐responsive units, DNA assembly‐based stimuli‐responsive systems have been applied in nanofabrication of 1D, 2D, and 3D DNA architectures, including the pure DNA architectures and DNA‐guided hybrid DNA architectures, which greatly enriches the DNA nanostructure library and provides abundant materials for later DNA‐based bioimaging, biotherapy and so on. Noteworthy, DNA assembly‐based stimuli‐responsive systems have also been applied to the construction of DNA nanomachines and constitutional dynamic networks that mimic mechanical functions and dynamic interactions in nature, respectively. In addition, a series of biosensors targeting metal ions, nucleic acids, proteins, small molecules, pH values, temperature and so on have been developed based on DNA assembly‐based stimuli‐responsive systems, which lay an important foundation for their application in biomedical research. More importantly, the DNA assembly‐based multi‐stimuli‐responsive systems, which can perform many functions at once, are starting to come into focus. They have shown great performance in bioimaging (fluorescence imaging, MR imaging) and therapeutics (chemotherapy, PTT, PDT, and gene therapy).

This review emphasized the applications of DNA assembly‐based stimuli‐responsive systems in DNA nanotechnology and discussed in nanofabrication and biomedical research. Beyond the contribution to the general field of dynamic DNA nanotechnology, these accomplishments also provide an excellent platform for the cross‐integration of chemistry, physics, biology, and other subjects. For example, DNA assembly‐based nanomachines that perform sensitive tests of physical quantities^[^ ^132^, ^379^, ^380^, ^381^ ^]^ or track biological behavior,^[^ ^380^ ^]^ have been emerging as valuable biophysical tools to investigate physical and biological mechanisms at the nanoscale or single‐molecule level.^[^ ^380^ ^]^ Furthermore, DNA assembly‐based stimuli‐responsive systems in logic gates^[^ ^272^, ^289^, ^338^, ^378^, ^382^ ^]^ and constitutional dynamic networks^[^ ^52^, ^239^, ^277^, ^278^, ^284^, ^285^, ^286^, ^287^, ^288^, ^383^, ^384^, ^385^, ^386^ ^]^ recently attract substantial research efforts, such orthogonal dynamic control provides additional dimensions for spatiotemporal control, and holds great application potential in the field of DNA computing, DNA information storage and biological interaction networks.

While DNA assembly‐based stimuli‐responsive systems have opened new frontiers in dynamic DNA nanotechnology, challenges and potential remain. We believe that breakthrough in any of the following challenges will give DNA assembly‐based stimuli‐responsive systems a better application prospects:

1) The development of new stimuli‐responsive units. Stimuli‐responsive units can sense the external physical or chemical stimuli and change states (including conformation, property, behavior, and so on) in prescribed ways. However, there is still a great room for expansion of the existing stimuli‐responsive units. For example, most stimuli‐responsive units perform transitions between only two specific states after sensing stimuli, and the types of stimuli‐responsive units based on similar mechanisms are still very limited, which greatly limits their application scenarios. Therefore, we expect to make breakthroughs in the mechanisms or numbers of stimuli‐responsive units. A database may be established for screening the simple and effective stimuli‐responsive units to meet their practical, customized needs. For example, the response accuracy and range of the triplex structure to pH values can be adjusted by sequence; therefore, it is feasible to construct complex stimuli response units for multi‐state transitions. In addition, the development of novel photosensitizers or photosensitive nanomaterials, temperature‐responsive polymers, and electrical‐responsive redox reactions can quantitatively expand the family of stimuli‐responsive units.
2) The optimization of DNA structural design, production costs, and research technique. With the assistance of a series of computer‐aided software (e.g., NUPACK, CaDNAno, CanDo), dynamic DNA nanotechnology has allowed for approaching of the complexity of natural structures, machines, and devices. The challenge here is to better balance the simplicity of design, the complexity of functionality, the high costs, and research technique. For example, an optimal design taking functionality into account can be obtained through more rigorous software. Advances in DNA synthesis can reduce costs of DNA molecules. Advanced techniques, such as cryo‐electron microscopy (cryo‐EM), all‐atom molecular dynamics (MD), and small angle X‐ray scattering (SAXS) can accurately reveal additional characteristics of dynamic DNA nanostructures. This requires the joint efforts in many fields, including chemistry, biology, physics, computer science, engineering, etc. Along with multi‐disciplinary efforts, the application prospect of dynamic DNA nanotechnology is inestimable.
3) The development of new way to integrate DNA assemblies and stimuli‐responsive units. The integration of DNA assemblies and stimuli‐responsive units is the key and foundation for the DNA assembly‐based stimuli‐responsive systems. At present, the integration of DNA and non‐DNA stimuli‐responsive units usually requires complex modification and purification processes, which is expensive in time and price. Notably, it has been recently reported that non‐DNA molecular systems that can dynamically control the DNA by directly mixing with the DNA system in accordance with the electrical, dynamic, and thermodynamic properties of the DNA itself. It is believed that with the deepening of the research of DNA properties and stimuli‐responsive units, more universal free molecular stimuli‐responsive systems can be developed to realize the macrodynamic regulation of DNA systems and to provide more space for the selection of DNA sequences and lengths. For example, the conformation or function of negatively charged DNA can be regulated by metal cations or positively charged polymers, as well as the assembly and dissociation of DNA nanostructures can be regulated by the protonation and deprotonation of pH‐responsive organic molecules.
4) The performance management of DNA assembly‐based stimuli‐responsive systems. When designing DNA assembly‐based stimuli‐responsive systems, it is necessary to consider their different requirements for response performance for specific application scenarios. Sensitivity, accuracy, timeliness, reversibility, versatility, and synergism are all very important considerations. For example, accuracy is one of the most important factors to ensure the assembly of nanostructures, which can be compensated at the expense of sensitivity and timeliness. The time period for the assembly of a complex 3D nanostructure usually takes days. The most important performance of DNA nanomachines is sensitivity and reversibility to achieve their physical or biological functions. In addition, for biomedical applications, sensitivity, versatility, and timeliness are critical for biosensing, and synergism can provide valuable information for combination therapy. Therefore, the rational design and combination of stimuli‐responsive units with different response performance will have a profound impact on promoting the application of DNA assembly‐based stimuli‐responsive systems.
5) The application in vivo. Dynamic DNA nanotechnology offers unique opportunities for in vivo bioimaging and therapy due to precise programmability and dynamic responsiveness of DNA based dynamic nanostructures. In vivo application of dynamic DNA nanotechnology requires careful consideration of additional factors. On the one hand, the side effects of dynamic DNA nanostructures on living bodies has to be overcome, such as biological toxicity of external stimuli, so as to ensure that there will be no irreversible serious damage to the living body in the process. On the other hand, the influence of living organisms on dynamic DNA nanostructures needs to be taken into account. For example, DNA nanostructures have to enter the cell through the natural barrier of the cell membrane, escape the degradation of ribozyme, and remain stable in the cycle process to achieve the ideal therapeutic effect. The vision of applying dynamic DNA nanotechnology to clinical therapy is still in its infancy, but the potential of DNA assembly‐based stimuli‐responsive systems applications should not be underestimated for the following reasons: 1) Direct external stimuli such as light, heat, and electricity are remotely controllable, and consequently DNA assembly‐based stimuli‐responsive systems have great application potential in light, heat and electrodynamics therapy. 2) Organisms have temperature, pH and ion regulatory systems and DNA exhibit superior biocompatibility, therefore, the corresponding DNA assembly‐based stimuli‐responsive systems can be applied in vivo. 3) The DNA assembly‐based stimuli‐responsive systems can coordinate with the complex biological system to realize the multi‐channel regulation of the biological systems.

All in all, DNA assembly‐based stimuli‐responsive systems have shown great potential in the field of nanofabrication, biomedical application, biocomputing, information storage, bionic network, etc. The increasing abundance of stimuli‐responsive unit libraries should allow the design and fabrication of dynamic DNA systems with high controllability to break the bottlenecks of static DNA nanotechnology.

## Conflict of Interest

The authors declare no conflict of interest.
